# MRI Diagnosis of Needle Tract Tumor Seeding Following Core Biopsy of Mucinous Carcinoma of the Breast

**DOI:** 10.7759/cureus.14493

**Published:** 2021-04-15

**Authors:** Botond K. Szabo, Akinyede Ojo, Dhafir Al-Okati

**Affiliations:** 1 Radiology, Barking, Havering and Redbridge University Hospitals NHS Trust, London, GBR; 2 Surgery, Barking, Havering and Redbridge University Hospitals NHS Trust, London, GBR; 3 Pathology, Barking, Havering and Redbridge University Hospitals NHS Trust, London, GBR

**Keywords:** breast cancer, breast mri, tumor seeding, core needle biopsy

## Abstract

Displacement or seeding of malignant cells into the needle tract following percutaneous biopsy is a known phenomenon, although it does not affect disease recurrence or overall survival of patients with breast cancer. It has, however, been previously hypothesized that needle tract seeding may occasionally progress to clinical tumor recurrence, and there have been case reports of breast cancer recurrence that are likely to be related to needle tract seeding. We are presenting a case of invasive mucinous carcinoma of the breast with associated malignant cell seeding within the biopsy tract, which was diagnosed preoperatively on contrast-enhanced MR imaging. A needle tract can often be visualized on contrast-enhanced MRI post biopsy and these changes may reflect tissue damage and regeneration only. In our case, an unusual nodular enhancement pattern was demonstrated along the biopsy needle tract, which was consistent with the histopathological finding of tumor seeding.

## Introduction

Percutaneous image-guided core biopsy is an established diagnostic tool for breast abnormalities with radiological appearances indeterminate or suspicious for malignancy [1]. Displacement or seeding of malignant cells into the needle tract following percutaneous biopsy is a known phenomenon, although it does not affect disease recurrence or overall survival of patients with breast cancer [2]. It has, however, been previously hypothesized that needle tract seeding may occasionally contribute to tumor recurrence [3], and reports of breast cancer recurrence that are likely to be related to needle tract seeding exist [4-7].

We are presenting a case of invasive mucinous carcinoma of the breast with associated malignant cell seeding within the biopsy tract, which was diagnosed preoperatively on contrast-enhanced MR imaging.

## Case presentation

**Figure 1:**
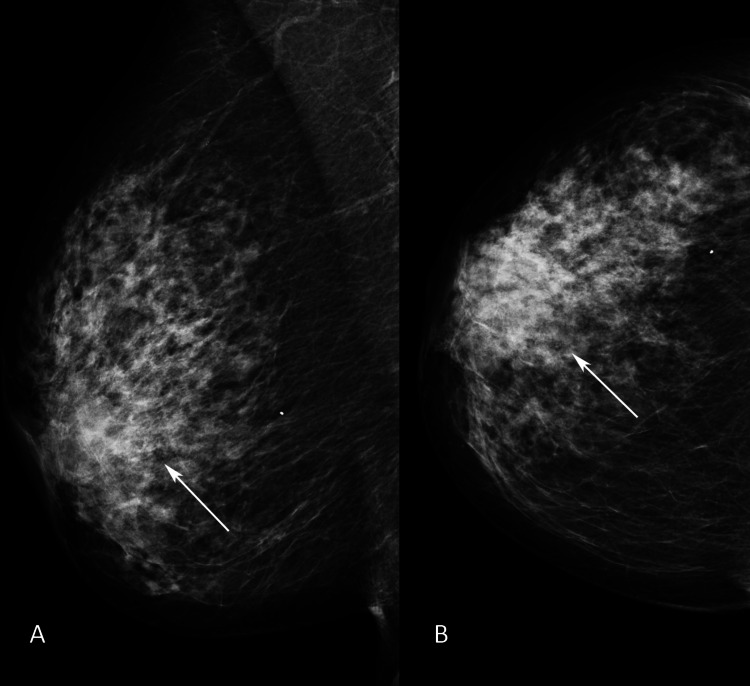
Standard mammographic views of the symptomatic right breast (A) Mediolateral oblique (MLO) and (B) craniocaudal (CC) mammographic views of the symptomatic right breast show dense glandular breast parenchyma with an asymmetric density in the sub-areolar area (arrows).

**Figure 2:**
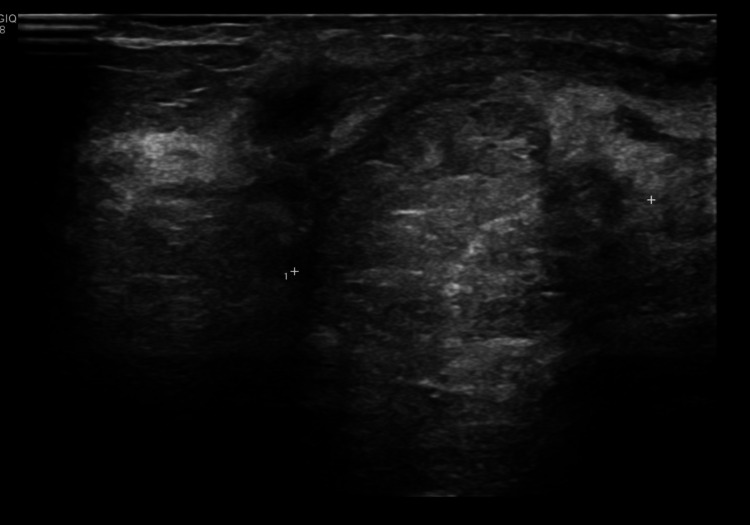
Ultrasound image of the palpable mass prior to biopsy Ultrasound image of the palpable peri-areolar mass at 12 o’clock, mostly isoechoic to surrounding breast tissue, heterogeneous internal echotexture and lobulated contours (biopsy proven invasive pure mucinous carcinoma).

**Figure 3:**
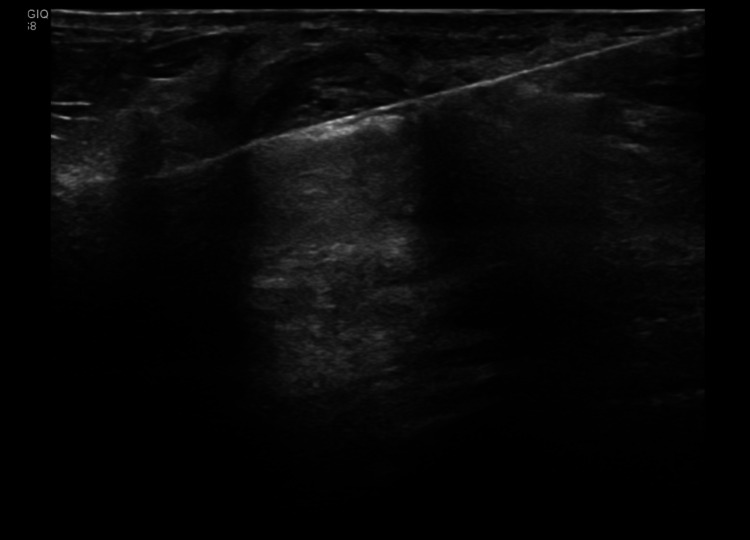
Ultrasound image of the palpable mass during diagnostic core biopsy Ultrasound image demonstrating the biopsy needle, 14G core biopsy samples were taken with three passes from medio-lateral approach.

**Figure 4:**
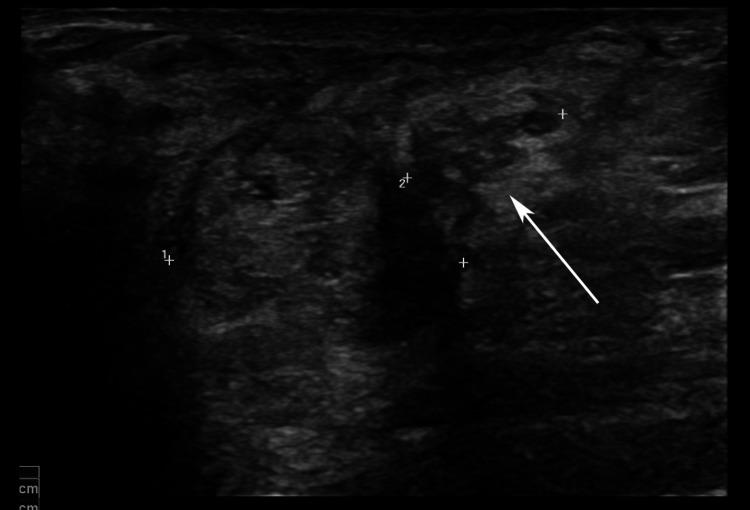
Ultrasound image 12 days after percutaneous core biopsy Ultrasound image 12 days after the biopsy showed two separate masses: the main mass at 12 o’clock and a further adjacent nodule at 2 o’clock in the sub-areolar location. A new 12-mm heterogeneous, cone-shaped nodule contiguous with the main mass was pointing medially and towards the skin (arrow).

**Figure 5:**
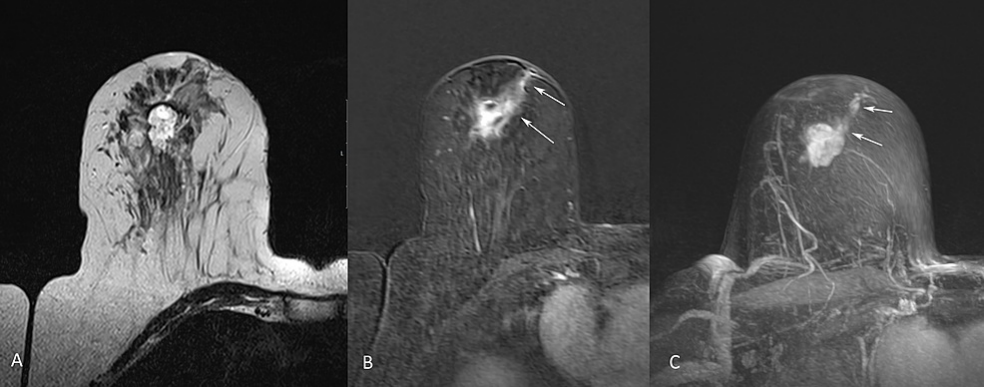
MR images of the right breast (A) Pre-contrast T2-weighted MR image of the right breast shows a high signal intensity mass in the retro-areolar area in keeping with the biopsy-proven invasive mucinous carcinoma. (B) Axial and (C) maximum intensity projection (MIP) contrast-enhanced subtracted images of the right breast show the enhancing index mass with nodular enhancement along the biopsy needle tract (arrows).

**Figure 6:**
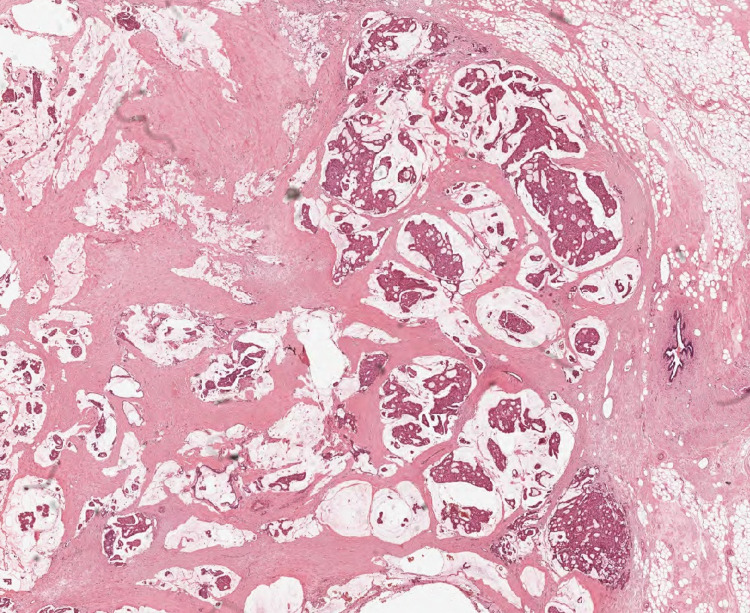
Hematoxylin and eosin stain. Right breast surgical specimen: main mass - high power (40x) Invasive mucinous carcinoma showing islands of malignant cells floating in pools of mucin.

**Figure 7:**
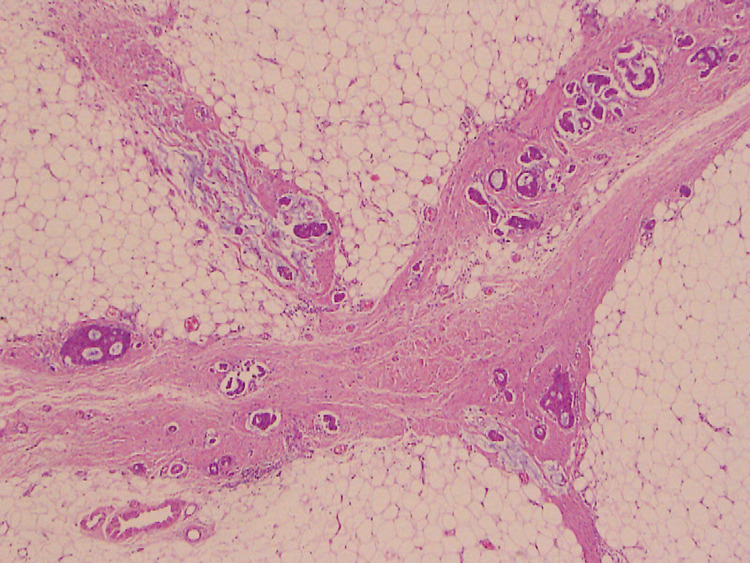
Hematoxylin and eosin stain. Right breast surgical specimen - high power (100x) Tumor spread along the needle track is seen, forming sheets of enlarged cells, with extracellular mucin production.

**Figure 8:**
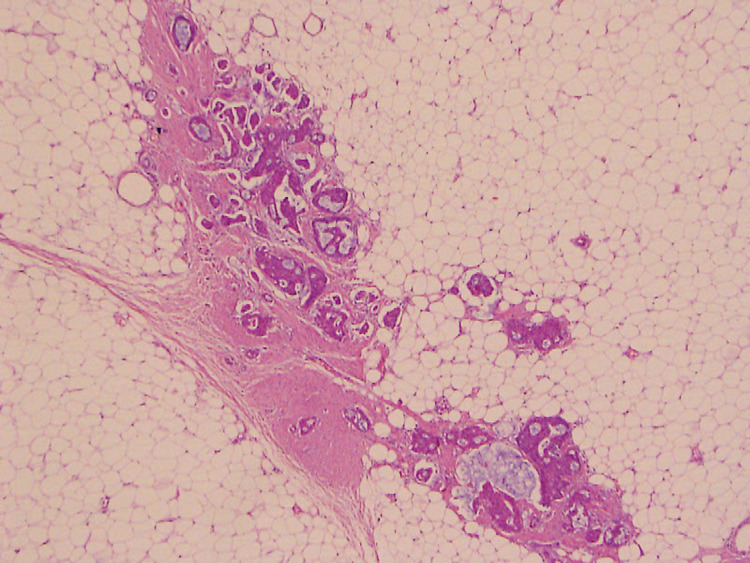
Hematoxylin and eosin stain. Right breast surgical specimen - high power (100x) Needle track is visualized with displacement of the same tumor cells, invading fibrous stroma and surrounding fat.

A 71-year-old female patient was presented with an approximately 3 cm peri-areolar mass in the 12 o’clock position in her right breast on physical examination, with no nipple retraction, skin dimpling or palpable axillary lumps. Bilateral mammograms showed very dense breast tissue with a suggestion of an asymmetric density in the right retro-areolar area. It was however difficult to characterize the lesion due to increased breast density (Figures 1A, 1B). Subsequent ultrasound examination of the whole right breast revealed a 26-mm ill-defined, somewhat heterogeneous, lobulated mass in the area of the palpable abnormality, mostly isoechoic to surrounding normal breast parenchyma. Ultrasound-guided core biopsy of the palpable mass was performed using a 14-gauge needle with three passes from the medio-lateral approach (Figures 2, 3). Histopathology showed invasive mucinous carcinoma of the breast and the diagnosis was given to the patient 12 days after the biopsy. At the second visit, two separate masses were felt on physical examination, one mass in the 12 o’clock and a further adjacent hard nodule at 2 o’clock in the sub-areolar location. A second ultrasound scan was performed, which showed a new 12-mm heterogeneous, cone-shaped nodule contiguous with the main mass pointing medially and towards the skin (Figure 4). Since the new palpable abnormality developed following the biopsy and aligned to the expected location of the biopsy needle tract, post biopsy organizing hematoma was suspected with a differential diagnosis of tumor displacement and growth in the biopsy tract. Dynamic contrast-enhanced MRI was performed to determine disease extent and to exclude additional lesions and tumor growth in the biopsy needle tract. The main tumor mass measured 25 mm which exhibited predominantly type 2 (plateau) time-signal intensity curves. Linear enhancement was demonstrated medial to the mass in keeping with the biopsy tract. Although this vague linear enhancement showed type 1 (steadily increasing) kinetics, there were two separate enhancing nodules along the tract measuring 10 mm and 8 mm with type 2 kinetics (Figures 5A-5C). A diagnosis of tumor seeding and/or displacement within the biopsy needle tract was made prior to surgery. Central wide local excision with removal of the biopsy tract and sentinel lymph node biopsy was performed. Histopathological examination of the excision specimen showed a 32-mm pure type of invasive mucinous cancer with tumor deposits within the biopsy needle tract measuring up to 6 mm (Figures 6-8). The whole size of tumor was 40 mm. One sentinel node was excised, which was involved by metastatic carcinoma. Due to involvement of sentinel lymph node, axillary dissection was done as second operation.

## Discussion

Previous studies investigated the incidence of tumor cell displacement along the biopsy needle tract in surgical specimens from patients with breast cancer, which showed that tumor seeding is present in 22%-50% of cases [2,8-10]. The risk of needle tract seeding could even be higher; a study examining the core needle wash following breast cancer biopsies showed a 65% risk [11]. The frequency of needle tract seeding found in surgical specimens inversely correlates with the time elapsed between the core biopsy and surgical excision, which suggests that most tumor cells do not survive displacement [9]. The literature shows that needle tract seeding accounts for seven percent of all breast cancer recurrence, and adjuvant therapy, including radiotherapy, has a protective effect [3,12]. Tumor recurrence that is linked to possible needle tract seeding is often seen in patients who did not receive radiotherapy [7]. Certain histological types are more frequently associated with tumor seeding such as invasive ductal carcinoma and DCIS, whereas it is significantly less likely in invasive lobular carcinomas. The technique used for breast lesion biopsy can also influence the risk of needle tract seeding. Multiple needle passes seem to increase the possibility of tumor seeding [11]. It has been shown that a vacuum-assisted biopsy device is less likely to cause tumor cell displacement than an automated spring-loaded core biopsy device [10].

It has been widely accepted that tumor seeding along the needle tract causes no increased recurrence rate when patients received appropriate adjuvant treatment [3,13]. There are, however, a number of case reports suggesting that needle tract seeding can progress to clinical breast cancer recurrence. Although invasive mucinous breast cancer is a rare histological type [14], three cases of needle tract seeding have been reported in the literature following diagnostic percutaneous core biopsy [5,6,12]. One report suggested that the incidence of needle tract seeding could be higher in mucinous cancer than other types of breast cancer, as intercellular adhesion is implicated in the process [4].

Ishizuna et al. reported a case similar to ours, where the biopsy tract was visible on preoperative breast MRI and the authors hypothesized that enhancement along the biopsy tract could indicate tumor seeding [6]. A recent study on vacuum-assisted biopsy related changes on MRI showed that the biopsy tract is visible in nearly 20% of the cases, and a thin faint contrast enhancement is also seen in half of them [15]. A needle tract can often be visualized on contrast-enhanced MRI and these changes may reflect post-biopsy tissue damage and regeneration only. In our case, an unusual nodular enhancement pattern was demonstrated along the biopsy tract, which was consistent with the histopathological finding of tumor seeding. Further studies should be conducted to determine whether different patterns of needle tract enhancement on MRI correlate with tumor cell displacement.

## Conclusions

Tumor cell seeding in the needle tract following percutaneous core biopsy of breast cancer can rarely progress into clinical disease recurrence. If there is, however, a clinical suspicion, contrast-enhanced MRI of the breast could be the imaging method of choice to detect tumor seeding in the biopsy needle tract, even preoperatively.

In case of typical radiological presentation of mucinous carcinoma, we should consider using a vacuum-assisted biopsy device, trocar technique or fewer needle passes with a regular spring-loaded biopsy approach to minimize the risk of tumor seeding.

## References

[1] O'Flynn EA, Wilson AR, Michell MJ (2010). Image-guided breast biopsy: state-of-the-art. Clin Radiol.

[2] Liebens F, Carly B, Cusumano P, Van Beveren M, Beier B, Fastrez M, Rozenberg S (2009). Breast cancer seeding associated with core needle biopsies: a systematic review. Maturitas.

[3] Loughran CF, Keeling CR (2011). Seeding of tumour cells following breast biopsy: a literature review. Br J Radiol.

[4] Yoneyama K, Nakagawa M, Hara A (2020). Local recurrence of breast cancer caused by core needle biopsy: case report and review of the literature. Int J Surg Case Rep.

[5] Harter LP, Curtis JS, Ponto G, Craig PH (1992). Malignant seeding of the needle track during stereotaxic core needle breast biopsy. Radiology.

[6] Ishizuna K, Ota D, Okamoto J (2011). A case of mucinous carcinoma of the breast in which needle tract seeding was diagnosed by preoperative diagnostic imaging. Breast Cancer.

[7] Chao C, Torosian MH, Boraas MC, Sigurdson ER, Hoffman JP, Eisenberg BL, Fowble B (2001). Local recurrence of breast cancer in the stereotactic core needle biopsy site: case reports and review of the literature. Breast J.

[8] Hoorntje LE, Schipper ME, Kaya A, Verkooijen HM, Klinkenbijl JG, Borel Rinkes IH (2004). Tumour cell displacement after 14G breast biopsy. Eur J Surg Oncol.

[9] Diaz LK, Wiley EL, Venta LA (1999). Are malignant cells displaced by large-gauge needle core biopsy of the breast?. AJR Am J Roentgenol.

[10] Michalopoulos NV, Zagouri F, Sergentanis TN (2008). Needle tract seeding after vacuum-assisted breast biopsy. Acta Radiol.

[11] Uematsu T, Kasami M (2008). Risk of needle tract seeding of breast cancer: cytological results derived from core wash material. Breast Cancer Res Treat.

[12] Thurfjell MG, Jansson T, Nordgren H, Bergh J, Lindgren A, Thurfjell E (2000). Local breast cancer recurrence caused by mammographically guided punctures. Acta Radiol.

[13] Fitzal F, Sporn EP, Draxler W (2006). Preoperative core needle biopsy does not increase local recurrence rate in breast cancer patients. Breast Cancer Res Treat.

[14] Chopra S, Evans AJ, Pinder SE, Yeoman LJ, Ellis IO, Elston CW, Wilson AR (1996). Pure mucinous breast cancer-mammographic and ultrasound findings. Clin Radiol.

[15] D’Angelo P, Marcon M, Linda A (2016). Pre-operative MR imaging in patients with DCIS: impact of VAB procedure-related changes. ECR.

